# Do podcasts improve the learning experience of dyslexic medical students?

**DOI:** 10.1192/j.eurpsy.2021.1584

**Published:** 2021-08-13

**Authors:** C. Parsons

**Affiliations:** Medical School, University of Bristol, Bristol, United Kingdom

**Keywords:** podcast, learning difficulty, widening participation, Dyslexia

## Abstract

**Introduction:**

There has been a rapidly increasing rate of students disclosing specific learning difficulties in higher education- in 2016 dyslexic students accounted for up to 5% of the student population.(Ryder D, Norwich B.)

**Objectives:**

It is important that provisions for alternative learners are developed in order to increase accessibility to medicine. Podcasts are an inexpensive, accessible and convenient method to both deliver education and interest pieces to a new generation of learners. This poster aims to explore the idea of harnessing the technology available to us to create an accessible, enjoyable platform to improve the experience of students with learning difficulties.

**Methods:**

A literature search reviewing the past and present provisions for students with learning difficulties was conducted using a range of databases covering educational, scientific and medical backgrounds. 315 papers were found across the databases, each analysed for relevance and 25 were selected as appropriate.

**Results:**

identified 5 key themes; the lack of awareness and importance of education, the power of audio learning, the practicalities of podcasts, adjustments to examinations specifically and finally additional provisions which accompany audio learning to create an all-inclusive educational experience. Altogether suggesting podcasts have an improved outcome for students with learning difficulties.

**Conclusions:**

It has been evidenced understanding is key to maximising learning potential and highlighted need to increase awareness of dyslexic needs in higher education institutions, generate audio centred provisions in conjunction to traditional materials and be aware of alternative provisions to cater for the spectrum of dyslexic needs.

